# Timing of risk factors, prodromal features, and comorbidities of dementia from a large health claims case–control study

**DOI:** 10.1186/s13195-024-01662-x

**Published:** 2025-01-16

**Authors:** Stefan Teipel, Manas Akmatov, Bernhard Michalowsky, Steffi Riedel-Heller, Jens Bohlken, Jakob Holstiege

**Affiliations:** 1 German Center for Neurodegenerative Diseases (DZNE) Rostock/Greifswald, Gehlsheimer Str. 20, Rostock, 18147, Germany; 2 Department of Psychosomatic Medicine, University Medicine Rostock, Gehlsheimer Str. 20, Rostock, 18147 Germany; 3 Department of Epidemiology and Healthcare Atlas, Central Research Institute of Ambulatory Health Care in Germany, Berlin, Germany; 4 German Center for Neurodegenerative Diseases (DZNE), Rostock/Greifswald, Greifswald, Germany; 5 Institute of Social Medicine, Occupational Health and Public Health, Faculty of Medicine, University of Leipzig, Leipzig, Germany

**Keywords:** Risk trajectory, Cancer, Hypertension, Behavioral disorders, Odds ratio, Dementia incidence, Health claims data

## Abstract

**Background:**

Many risk factors for dementia have been identified, but the timing of risk is less well understood. Here, we analyzed risk factors in a case–control study covering 10 years before an incident dementia diagnosis.

**Methods:**

We designed a case–control study using insurance claims of outpatient consultations of patients with German statutory health insurance between January 1, 2012, and December 31, 2022. We included patients with an incident diagnosis of dementia and controls without a diagnosis of dementia matched 1:2 for age, sex, region, and earliest year of outpatient encounter. We selected exposures based on previous systematic reviews, case–control and cohort studies reporting on risk factors, comorbidities, and prodromal features of dementia. We calculated the prevalence of risk factors in cases and controls and odds ratios for each year before the index date, along with Bonferroni-corrected confidence intervals, using conditional logistic regression.

**Results:**

We identified a total of 1,686,759 patients with incident dementia (mean (SD) age, 82.15 (6.90) years; 61.70% female) and 3,373,518 matched controls (mean (SD) age, 82.15 (6.90) years; 61.70% female). Study participants were followed up for a mean (SD) of 6.6 (2.3) years. Of the 63 risk factors and prodromal features examined, 56 were associated with an increased risk of dementia in all years during the 10th and the 1st year before the index date. These included established risk factors, such as depression, hypertension, hearing impairment, nicotine and alcohol abuse, obesity, hypercholesterolaemia, traumatic brain injury, and diabetes. The greatest risk, with odds ratios greater than 2.5, was conferred by delirium, memory impairment, mental retardation, personality and behavioral disorders, sensory disorders, schizophrenia, and psychosis. Cancer was associated with a reduced risk of dementia.

**Conclusions:**

This large case–control study confirmed established risk factors of dementia. In addition, the study identified non-specific diagnoses that showed a steep increase in risk close to the index date, such as psychosis, conduct disorder, and other sensory disorders. Consideration of these diagnoses, which may represent prodromal features rather than risk factors for dementia, may help to identify people with dementia in routine care.

**Supplementary Information:**

The online version contains supplementary material available at 10.1186/s13195-024-01662-x.

## Background

Dementia is a major public health concern worldwide [1] and is associated with a variety of conditions that affect brain function [2]. At age 50 and older, the leading cause of dementia are neurodegenerative diseases, with Alzheimer's disease (AD) being the most common cause of dementia, followed by Lewy body disease and frontotemporal lobar degeneration [3]. With increasing age, cerebrovascular disease becomes an increasingly important cofactor for the manifestation of dementia in combination with neurodegenerative disease [4]. Rarer forms of dementia are caused by rare neurodegenerative diseases such as Huntington's disease, metabolic diseases such as hypothyroidism, or chronic intoxication such as alcohol abuse [5, 6]. The broad spectrum of underlying causes is reflected in the large number of risk factors that have been identified. In addition, dementia may be associated with comorbid conditions such as Parkinson's disease [7] or may be preceded by prodromal features such as mild cognitive impairment or general frailty [8]. The transition between risk factors, prodromal features, and comorbidities is fluid, so late-onset depression is regarded as both a risk factor and a prodromal feature of dementia [9], and delirium can be a risk factor, a prodromal feature, or a comorbidity [10]. Therefore, these categories should be seen as a pragmatic approach to navigating within a broad range of factors that do not capture the full complexity of interactions.

Two consortia, led by the Lancet Commission [11, 12] and the World Health Organization [13], have identified a core set of risk factors for dementia, listed in Table 1, including hypertension, depression, and hearing loss. Numerous other risk factors include conditions that directly affect brain function, such as delirium [14], cerebrovascular disease [15, 16], and psychiatric conditions, such as anxiety [17] or bipolar disorder [18]. Other known risk factors include sleep disorders [19, 20], migraine [21], autonomic dysfunction [22], pain [23], cardiovascular disease [24], sensory impairment [25], infections [26], and gastrointestinal disorders [27]. Comorbidities include Parkinson’s disease [7] and schizophrenia [28]. Risk factors have been identified based on several types of sources, including large-scale prospective cohorts on the prevalence and incidence of dementia reviewed in [29], retrospective cohort studies [30, 31], and case–control studies based on health claims data [23, 32, 33].

**Table 1:** Risk factors for dementia identified by the Lancet Commission and the World Health Organization

Risk factor	Source
Less education	LC
Hearing loss^*^	LC
Traumatic brain injury^*^	LC
Hypertension^*^	LC, WHO
Alcohol^*^	LC, WHO
Obesity^*^	LC, WHO
Smoking^*^	LC, WHO
Depression^*^	LC, WHO
Social isolation	LC, WHO
Physical inactivity	LC, WHO
Diabetes^*^	LC, WHO
Air pollution	LC
Visual impairment^*§^	LC
Dyslipidemia^*§^	WHO
Nutrition	WHO

*LC* Lancet Commission [12]**,** *WHO* World Health Organization [13]

^*^Accessible by health claims data

^§^Added in the 2024 Update of the Lancet Commission report [11]

Here, we aimed to extend previous evidence by including a very broad range of potential risk factors, comorbidities, and prodromal features in a case–control study based on a large health claims database representing documented diagnoses in general and specialized physician practices. We included more than 1.6 million dementia cases and more than 3.2 million age- and sex-matched controls covering 10 years before the index date. These data provide high power to determine the temporal trajectories of risks over 10 years preceding an incident-documented dementia diagnosis in physician practices.

## Methods

### Study design

We designed a case–control study to identify diseases and signs/symptoms that were diagnosed up to 10 years before the onset of dementia. This study was based on nationwide outpatient claims data of all individuals with statutory health insurance (SHI) from 2012 to 2022. The assignment of quarters to years and time intervals is described in Supplementary Table 1. The SHI covers almost 88% of the total German population, corresponding to a population of 73,109,811 individuals in 2022. The dataset comprises diagnoses from outpatient general and specialist practices that were coded using the German modification of the 10th edition of the International Classification of Diseases and Related Health Problems (ICD-10-GM), along with diagnostic certainty (e.g., “confirmed” or “excluded”) and demographic characteristics of insurees (sex, age, and region of residence) who visited a SHI-authorized physician at least once in the respective years. The use of claims data for scientific research in Germany is regulated by the Code of Social Law (Sozialgesetzbuch, SGB V). Ethical approval and informed consent are not required for routinely collected pseudonymized data. This study followed the Strengthening the Reporting of Observational Studies in Epidemiology (STROBE) reporting guidelines [34].

The claims dataset has already been used to study early risk factors and comorbidities as well as prodromal features for various chronic conditions. This regards the identification of novel associations as well as the reproduction of known risk factors and comorbidities observed in former studies. The latter include but are not limited to smoking as a protective factor for Parkinson’s disease [35], obesity as a risk factor for childhood multiple sclerosis (MS), high health care utilization in the five years before the first diagnosis of MS in affected patients [36] and bi-directional associations of diabetes and depression [37].

Cases of newly diagnosed dementia and controls (not diagnosed with dementia) were identified in a cohort of patients ≥ 65 years of age (*n* = 15,891,157 in 2022) with outpatient visits in the respective year and at least 1 time 3 years before the index year or earlier without a dementia diagnosis preceding the index year. Incident cases were those receiving the first “confirmed” diagnosis of dementia (ICD-10-GM-codes = ‘F01’, ‘F02’,’F03’,’G30’) in one quarter between 2015 and 2022 and at least one additional diagnosis in one of the following three quarters. Controls without a dementia diagnosis were matched to each case (2:1) by sex, age at disease onset (in years) and the first calendar year of an outpatient visit in the dataset.

### Diagnostic categories

The diagnostic categories and corresponding ICD-10 codes are shown in Table 2. We defined the diagnostic categories and ICD-10-GM-codes based on a literature review and clinical expert opinion. We included risk factors previously identified by the international consortia of the Lancet Commissions [12] and the World Health Organization [13]. We also referred to an earlier study by our group that identified risk factors and prodromal features of Parkinson's disease [35]. We categorized factors into risk factors, prodromal features and comorbidities. Risk factors were defined as conditions that have been previously treated as risk factors in the literature and were found to generally precede the onset of dementia by several years. Typical examples are hypertension, alcohol abuse, hearing impairment, and diabetes mellitus type II [12]. Prodromal features of dementia are cognitive impairment, memory impairment and frailty/senility. A typical comorbidity is Parkinson’s disease. We acknowledge that the distinction between these categories is not very clear. For example, depression is considered both a risk factor and a prodromal feature [9]. Therefore, we use these categories for pragmatic structuring of a large number of factors rather than as a clear-cut categorization system.

**Table 2:** Risk factors, prodromal or concomitant conditions and corresponding ICD codes

Diseases/conditions	ICD-10 codes
**Motor dysfunctions and injuries**	
Abnormalities of gait and mobility	R26
Tremor	G25.0, G25.1, G25.2, R25.1
Extremity injury	S42, S47, S48, S52, S57, S58, S62, S67, S68, S72, S77, S78, S82, S87, S88, S92, S97, S98
**Psychiatric disorders**	
Anxiety	F41.0, F41.1, F41.9
Bipolar disorder	F31
Cognitive impairment	U51
Conduct disorder	F91, F92
Delirium	F05
Depression	F32, F33
Memory impairment	F06.7, R41.3, R41.8
Mental retardation	F70, F71
Emotional state disorders	R45
Personality and behavioral disorders due to brain disease, damage and dysfunction	F07
Psychosis	F29
Schizophrenia	F20, F25
**Impairment of sensory organs**	
Anosmia	R43.0
Visual impairment	H53
Blindness	H54
Hearing impairment, bilateral	H90.0, H90.3, H90.6
Hearing impairment, unilateral	H90.1, H90.4, H90.7
Hearing impairment, side unspecified	H90.2, H90.5, H90.8, H91
Other sensory disorders	R44
**Sleep disorders**	
Insomnia	F51.0 with G47.0
Other sleeping disorder	F51.2, F51,8, F51.9, G47.2, G47.9
Restless legs syndrome	G25.81
Sleep apnea	G47.3
**Autonomic dysfunction and fatigue**	
Bladder disorder	N31
Constipation	K59.0
Dizziness	R42
Fatigue	G93.3
Hypotension	I95, R03.1
Sexual dysfunction	F52.0, F52.1, F52.2, F52.8, F52.9
**Risk factors**	
Obesity	E66
Alcohol abuse/dependence	F10.1, F10.2
Nicotine abuse/dependence	F17.1, F17.2
Traumatic brain injury	S06.0, S06.1. S06.2, S06.3
**Vascular Diseases and Metabolic Disorders**	
Atrial fibrillation	I48
Carotid artery stenosis	I65.2
Cerebrovascular disease	I60-I64, I65.0, I65.1, I65.3, I65.8, I65.9, I66-I69
Type 2 Diabetes mellitus	E11
Hypercholesterolaemia	E78.0
Hypertension	I10
Ischemic heart disease	I20-I25
**Infections**	
Acute upper respiratory infection	J06
Antibiotic resistance	U81
Cystitis	N30
Pneumonia	J12-J18
Sepsis	A41
**Gastrointestinal disorders**	
Crohns disease	K50
Duodenal ulcer	K26, K27
Gastritis	K29
Reflux disease	K21
Stomach ulcer	K25
Ulcerative colitis	K51
**Comorbidities**	
Epilepsy	G40
Multiple sclerosis	G35
Parkinsons disease	G20, G21, G22
Migraine	G43
**General symptoms & conditions**	
Cancer	C00-C97
Ostearthritis	M15-M19
Other rheumatoid arthritis	M06
Senility	R54
Unspecific pain	R52.1, R52.2, R52.9

After the completion of our analyses, the Lancet Commissions updated report 2024 [11] added visual impairment and LDL hypercholesterolaemia to the 2020 list of risk factors. These two factors were already included in our selection.

### Statistical analysis

Prevalence in cases and controls was calculated for risk factors, prodromal features, and comorbidities of dementia for every year from year one to year 10 before the index date. Conditional logistic regression was used to estimate Odds ratios (OR) for dementia according to prevalence of each factor, i.e. risk factors, prodromal features, and comorbidities of dementia for the entire observation time of ten years before the index date. In addition, OR were estimated for the periods 1 year, 2 to 4 years, and 5 to 10 years before the index date for each factor of interest. The 95% CIs for OR were conservatively corrected for multiple comparisons using the Bonferroni method. Statistical significance was assumed when the 95% CI of the OR did not overlap with the null value (e.g., OR = 1.0). Statistical analyses were performed using SAS, version 9.4 (SAS Institute).

## Results

A total of 1,686,759 patients with incident dementia (mean [SD] age, 82.15 (6.90) years; 61.70% female) between 2015 and 2022 and 3,373,518 matched controls (mean [SD] age, 82.15 (6.90) years; 61.70% female) were identified. The age range for cases and controls was 65 to 101 years. The demographic characteristics for each time period are given in Table 3. The mean (SD) follow-up time was 6.6 (2.3) years in both the cases and controls.

**Table 3:** Demographic characteristics

years before index date	N (cases)	% women (cases)	mean age (SD) [years] (cases)	N (controls)	% women (controls)	mean age (SD) [years] (controls)
−1 year	1,686,759	61.70%	82.15 (6.90)	3,373,518	61.70%	82.15 (6.90)
-(2 to 4) years	1,686,759	61.70%	82. 15 (6.90)	3,373,518	61.70%	82.15 (6.90)
-(5 to 10) years	1,373,002	61.59%	82.24 (6.88)	2,746,004	61.59%	82.24 (6.88)

**Fig. 1:**
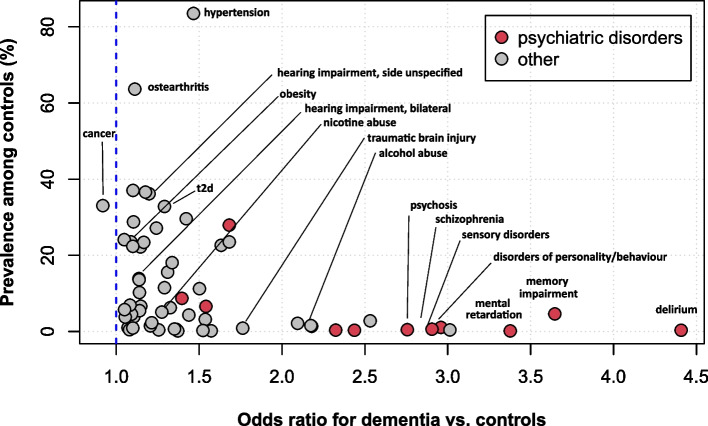
Prevalence in controls regressed on odds ratios. The graph plots the prevalence of each condition in the controls against the odds ratio for dementia risk. Annotated are the risk factors for dementia identified by the Lancet Commission and the World Health Organization (see Table 1). In addition, conditions with an OR > 2. were annotated. The interactive Supplementary Fig. 1 (online in Supplementary Material 2) allows identification of each individual condition in the graph. t2d – type 2 diabetes

**Fig. 2:**
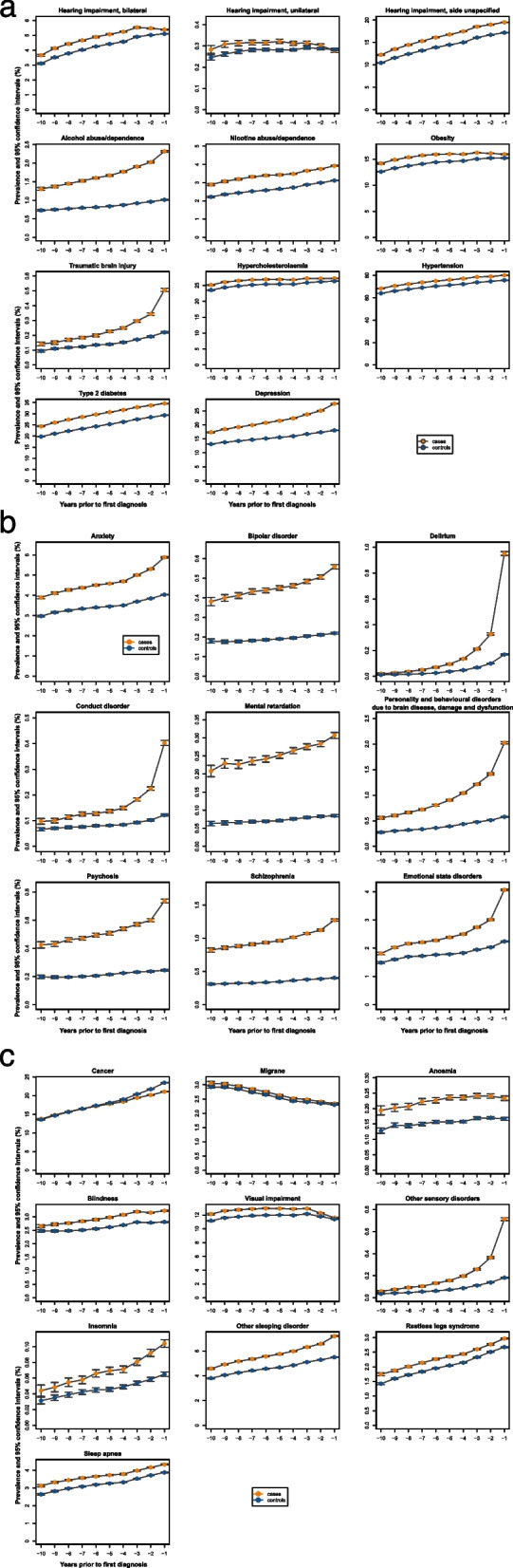
Prevalence of risk factors by year before the index date in cases and controls. **a** Prevalence of 2020 Lancet Commission and WHO risk factors. **b** Prevalence of psychiatric conditions. **c** Prevalence of cancer, migraine, impairments of sensory organs and sleep disorders. Prevalence of each condition (and 95% confidence intervals) by year before the index date in the cases and controls

**Fig. 3:**
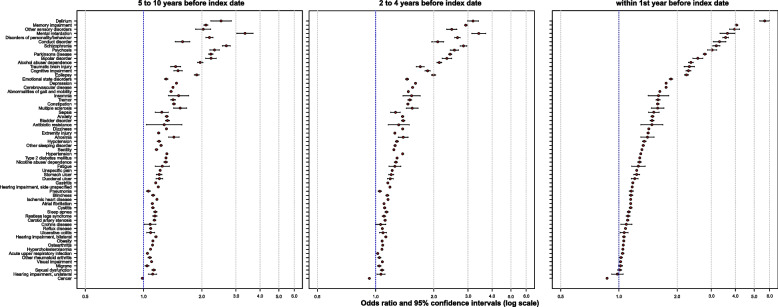
Odds ratios of all conditions in three time periods. The figure shows the odds ratios and their 95% confidence intervals for all conditions within the time periods 5 to 10 years, 2 to 4 years, and 1 year before the index date. The conditions are sorted in descending order by the size of the odds ratios in year 1 before the index date

Table 4 shows the proportion of control and index cases with a condition and the resulting odds ratios and their 95% Bonferroni corrected confidence intervals for each of the time intervals 1 year, 2 to 4 years, and 5 to 10 years. Figure 1 plots the prevalence of a condition among controls relative to its odds ratio (and in an interactive format in Supplementary Fig. 1 in Supplementary Material 2). Figure 2 and Supplementary Fig. 2 show the annual prevalence of conditions in index cases and controls from year −10 to year −1. Figure 3 shows the odds ratios and confidence intervals for the intervals 5 to 10 years, 2 to 4 years, and 1 year before the index date, sorted according to the size of the odds ratios.

**Table 4:** Odds ratios and 95% CIs adjusted for multiple comparisons of risk factors, prodromal features, and comorbidities in cases compared with controls in the year before index date and in the time periods 2 to 4 years and 5 to 10 years before the index date

Category	Disease/ condition	1 year			2–4 years			5–10 years		
		**Prevalence cases**	**Prevalence controls**	**OR (95%-CI)**	**Prevalence cases**	**Prevalence controls**	**OR (95%-CI)**	**Prevalence cases**	**Prevalence controls**	**OR (95%-CI)**
Motor dysfunctions and injuries	Abnormalities of gait and mobility	23.76	16.31	1.63 (1.61–1.64)	22.21	16.45	1.48 (1.46–1.49)	11.90	9.03	1.39 (1.37–1.41)
	Extremity injury	7.85	5.70	1.42 (1.40–1.44)	11.25	9.18	1.26 (1.24–1.27)	9.71	8.30	1.20 (1.18–1.21)
	Tremor	2.75	1.75	1.59 (1.55–1.63)	3.34	2.25	1.50 (1.47–1.53)	2.77	1.97	1.42 (1.39–1.46)
Psychiatric disorders	Anxiety	5.89	4.04	1.49 (1.47–1.51)	7.63	5.67	1.38 (1.36–1.40)	7.24	5.62	1.32 (1.30–1.34)
	Bipolar disorder	0.56	0.22	2.56 (2.41–2.71)	0.61	0.26	2.33 (2.21–2.47)	0.55	0.25	2.23 (2.10–2.38)
	Cognitive impairment	2.21	0.98	2.29 (2.22–2.35)	1.77	0.96	1.86 (1.80–1.91)	0.76	0.50	1.51 (1.43–1.58)
	Conduct disorder	0.40	0.12	3.31 (3.07–3.57)	0.36	0.17	2.09 (1.95–2.24)	0.25	0.16	1.59 (1.46–1.73)
	Delirium	0.95	0.17	5.66 (5.34–6.00)	0.50	0.16	3.19 (2.98–3.41)	0.16	0.06	2.51 (2.23–2.84)
	Depression	27.59	18.01	1.75 (1.74–1.77)	30.44	21.60	1.61 (1.59–1.62)	27.12	20.36	1.48 (1.46–1.49)
	Emotional state disorders	4.07	2.24	1.85 (1.82–1.89)	5.28	3.70	1.45 (1.43–1.48)	4.78	3.71	1.31 (1.28–1.33)
	Memory impairment	10.72	2.88	4.05 (3.99–4.11)	8.70	3.16	2.92 (2.88–2.97)	4.15	2.03	2.10 (2.05–2.15)
	Mental retardation	0.31	0.08	3.64 (3.33–3.97)	0.34	0.10	3.41 (3.14–3.70)	0.30	0.09	3.34 (3.04–3.68)
	Personality and behavioural disorders due to brain disease. damage and dysfunction	2.02	0.58	3.56 (3.44–3.68)	1.85	0.71	2.65 (2.56–2.74)	1.26	0.58	2.19 (2.10–2.28)
	Psychosis	0.74	0.24	3.03 (2.87–3.20)	0.81	0.32	2.56 (2.43–2.68)	0.71	0.31	2.33 (2.20–2.46)
	Schizophrenia	1.28	0.41	3.19 (3.06–3.32)	1.30	0.46	2.84 (2.73–2.96)	1.12	0.42	2.68 (2.55–2.80)
Impairment of sensory organs	Anosmia	0.23	0.17	1.40 (1.30–1.52)	0.44	0.31	1.39 (1.31–1.47)	0.48	0.33	1.43 (1.35–1.52)
	Visual impairment	11.61	11.41	1.02 (1.01–1.03)	20.47	19.19	1.08 (1.07–1.09)	22.45	20.88	1.10 (1.09–1.11)
	Blindness	3.23	2.81	1.15 (1.13–1.18)	4.81	4.22	1.15 (1.13–1.17)	5.10	4.58	1.12 (1.10–1.14)
	Hearing impairment. bilateral	5.40	5.10	1.06 (1.04–1.08)	10.04	9.00	1.13 (1.12–1.14)	9.58	8.40	1.16 (1.14–1.18)
	Hearing impairment. side unspecified	19.5	17.18	1.17 (1.16–1.18)	29.14	25.82	1.19 (1.18–1.20)	26.86	23.74	1.19 (1.18–1.20)
	Hearing impairment. unilateral	0.28	0.28	0.98 (0.92–1.05)	0.59	0.56	1.07 (1.02–1.12)	0.68	0.61	1.11 (1.06–1.17)
	Other sensory disorders	0.71	0.18	3.96 (3.73–4.20)	0.55	0.22	2.47 (2.33–2.62)	0.26	0.13	2.03 (1.86–2.22)
Sleep disorders	Insomnia	0.10	0.07	1.60 (1.42–1.81)	0.15	0.09	1.53 (1.39–1.70)	0.13	0.08	1.52 (1.35–1.71)
	Other sleeping disorder	7.20	5.50	1.34 (1.32–1.35)	9.51	7.71	1.26 (1.25–1.28)	8.92	7.40	1.23 (1.21–1.25)
	Restless legs syndrome	2.97	2.67	1.11 (1.09–1.14)	3.31	3.01	1.10 (1.08–1.13)	2.93	2.57	1.14 (1.12–1.17)
	Sleep apnea	4.33	3.88	1.12 (1.10–1.14)	4.95	4.39	1.14 (1.12–1.16)	4.59	4.03	1.15 (1.13–1.17)
Autonomic dysfunction and fatigue	Bladder disorder	3.39	2.31	1.48 (1.45–1.51)	4.07	2.97	1.39 (1.36–1.42)	3.30	2.51	1.33 (1.30–1.36)
	Constipation	6.83	4.43	1.59 (1.56–1.61)	9.68	6.81	1.47 (1.45–1.49)	7.56	5.42	1.44 (1.41–1.46)
	Dizziness	19.20	14.33	1.43 (1.41–1.44)	25.74	20.17	1.38 (1.37–1.39)	21.24	17.27	1.31 (1.30–1.33)
	Fatigue	0.21	0.17	1.26 (1.16–1.36)	0.29	0.23	1.26 (1.17–1.35)	0.24	0.20	1.25 (1.15–1.36)
	Hypotension	3.11	2.32	1.35 (1.32–1.38)	4.76	3.74	1.29 (1.27–1.31)	4.26	3.57	1.20 (1.18–1.23)
	Sexual dysfunction	2.46	2.44	1.01 (0.99–1.03)	3.58	3.34	1.08 (1.06–1.10)	4.04	3.63	1.13 (1.10–1.15)
Risk factors	Alcohol abuse/ dependence	2.32	1.01	2.35 (2.29–2.42)	2.44	1.18	2.13 (2.08–2.19)	2.09	1.09	1.96 (1.90–2.02)
	Nicotine abuse/ dependence	3.92	3.12	1.28 (1.25–1.30)	4.85	3.87	1.27 (1.25–1.30)	4.66	3.65	1.30 (1.28–1.33)
	Migraine	2.34	2.30	1.02 (1.00–1.04)	3.03	2.90	1.04 (1.02–1.07)	3.46	3.33	1.04 (1.02–1.07)
	Obesity	15.97	15.23	1.06 (1.05–1.07)	20.13	18.93	1.08 (1.07–1.09)	20.43	18.73	1.12 (1.11–1.13)
	Traumatic brain injury	0.51	0.22	2.31 (2.18–2.45)	0.72	0.43	1.70 (1.62–1.78)	0.54	0.37	1.46 (1.38–1.55)
Vascular diseases and metabolic disorders	Atrial fibrillation	21.17	18.99	1.15 (1.14–1.16)	20.16	18.64	1.10 (1.09–1.12)	14.61	13.39	1.11 (1.10–1.12)
	Carotid artery stenosis	7.13	6.47	1.11 (1.09–1.12)	8.63	7.78	1.12 (1.11–1.13)	7.50	6.67	1.14 (1.12–1.15)
	Cerebrovascular disease	24.58	15.71	1.75 (1.74–1.77)	25.67	18.24	1.55 (1.54–1.57)	20.30	15.29	1.42 (1.40–1.43)
	Hypercholesterolaemia	27.21	26.31	1.05 (1.04–1.06)	32.58	30.96	1.08 (1.07–1.09)	32.60	30.83	1.09 (1.08–1.10)
	Hypertension	80.08	75.69	1.30 (1.29–1.31)	83.92	79.20	1.38 (1.37–1.39)	80.36	75.82	1.32 (1.31–1.33)
	Ischemic heart disease	31.74	28.91	1.15 (1.14–1.16)	34.54	31.40	1.16 (1.15–1.17)	31.40	28.20	1.17 (1.16–1.18)
	Type 2 diabetes mellitus	34.62	29.28	1.28 (1.27–1.29)	35.78	30.18	1.29 (1.28–1.30)	32.55	27.02	1.31 (1.29–1.32)
Infections	Acute upper respiratory infection	5.42	5.21	1.04 (1.03–1.06)	13.19	12.91	1.03 (1.01–1.04)	14.99	14.44	1.05 (1.04–1.06)
	Antibiotic resistance	0.09	0.06	1.48 (1.30–1.69)	0.09	0.07	1.32 (1.16–1.49)	0.04	0.03	1.28 (1.04–1.58)
	Cystitis	4.58	4.01	1.15 (1.13–1.17)	8.78	7.97	1.11 (1.10–1.13)	9.10	8.26	1.12 (1.10–1.13)
	Pneumonia	2.24	1.94	1.16 (1.13–1.19)	3.81	3.63	1.05 (1.03–1.07)	3.25	3.08	1.06 (1.04–1.08)
	Sepsis	0.37	0.24	1.51 (1.42–1.61)	0.43	0.34	1.27 (1.20–1.34)	0.29	0.23	1.24 (1.15–1.34)
Gastrointestinal disorders	Crohns disease	0.30	0.27	1.09 (1.02–1.17)	0.36	0.33	1.06 (1.00–1.13)	0.36	0.33	1.08 (1.01–1.16)
	Duodenal ulcer	0.84	0.70	1.21 (1.16–1.26)	1.14	0.96	1.19 (1.15–1.23)	1.15	0.96	1.21 (1.16–1.25)
	Gastritis	12.43	10.74	1.18 (1.17–1.19)	17.69	15.68	1.16 (1.15–1.17)	18.5	16.46	1.16 (1.14–1.17)
	Reflux disease	15.37	14.38	1.08 (1.07–1.09)	18.55	17.37	1.08 (1.07–1.09)	18.31	17.10	1.09 (1.08–1.10)
	Stomach ulcer	1.23	1.00	1.23 (1.19–1.28)	1.73	1.44	1.20 (1.17–1.24)	1.70	1.42	1.20 (1.17–1.24)
	Ulcerative colitis	0.60	0.56	1.06 (1.02–1.12)	0.74	0.68	1.09 (1.05–1.14)	0.73	0.67	1.09 (1.04–1.14)
Comorbidities	Epilepsy	3.54	1.62	2.23 (2.18–2.28)	3.27	1.67	1.99 (1.95–2.04)	2.44	1.31	1.88 (1.83–1.94)
	Multiple sclerosis	0.28	0.18	1.58 (1.47–1.71)	0.31	0.20	1.55 (1.44–1.66)	0.30	0.20	1.55 (1.43–1.67)
	Parkinsons disease	5.82	2.18	2.78 (2.72–2.83)	5.26	2.24	2.43 (2.38–2.47)	3.46	1.59	2.23 (2.17–2.28)
General symptoms and conditions	Cancer	21.05	23.44	0.87 (0.86–0.88)	24.86	26.24	0.93 (0.92–0.94)	22.37	22.61	0.99 (0.98–0.99)
	Ostearthritis	45.70	44.46	1.05 (1.05–1.06)	55.67	53.64	1.09 (1.08–1.10)	54.33	51.85	1.11 (1.10–1.12)
	Other rheumatoid arthritis	3.86	3.78	1.02 (1.00–1.04)	4.69	4.49	1.05 (1.03–1.06)	4.57	4.26	1.08 (1.06–1.10)
	Senility	18.45	14.78	1.32 (1.30–1.33)	17.06	14.30	1.24 (1.23–1.25)	8.88	7.79	1.17 (1.15–1.18)
	Unspecific pain	20.75	17.54	1.24 (1.22–1.25)	23.17	19.91	1.22 (1.21–1.23)	17.57	15.01	1.22 (1.21–1.23)

### Risk factors from Lancet Commissions [12] and the WHO [13]

Five to 10 years before the index date, a diagnosis of depression was associated with an increased OR for dementia (OR, 1.45; 95% CI, 1.44–1.46), and the OR increased until 1 year before the index date (Table 4, Fig. 2a). Bilateral hearing impairment showed an increased OR for dementia 5 to 10 years before the index date (OR, 1.16; 95% CI, 1.14–1.17), with the effect decreasing until 1 year before the index date (OR, 1.06; 95% CI, 1.05–1.08). The effect was similar for hearing impairment with the side unspecified (OR, 1.18; 95% CI, 1.17–1.19 at −10 years, and OR, 1.17; 95% CI, 1.16–1.18 at −1 year). The OR for unilateral hearing impairment was smaller than that for bilateral or unspecified hearing impairment and was no longer significant at 1 year before the index date (OR, 0.99; 95% CI, 1.92–1.05) (Table 4, Fig. 2a). Alcohol abuse and traumatic brain injury (TBI) had an increased OR at 5 to 10 years before the index date (alcohol OR, 1.96; 95% CI 1.90–2.02; TBI OR, 1.49; 95% CI 1.40–1.57), with a further increase until 1 year before the index date (alcohol OR, 2.34; 95% CI 2.28–2.41; TBI OR 2.30; 95% CI, 2.17–2.43) (Table 4, Fig. 2a).

Nicotine abuse, obesity, and diabetes mellitus type 2 had an increased OR for dementia 10 years before the index date (nicotine OR, 1.30; 95% CI, 1.27–1.32; obesity OR, 1.11; 95% CI, 1.10–1.12; diabetes: OR, 1.31; 95% CI, 1.29–1.32), with a slight decrease in the effect until year 1 before the index date (nicotine: OR, 1.28; 95% CI, 1.26–1.31; obesity OR, 1.06; 95% CI, 1.05–1.07; diabetes: OR, 1.28; 95% CI, 1.27–1.29) (Table 4, Fig. 2a).

Hypertension had an increased OR 5 to 10 years before the index date (OR, 1.31; 95% CI, 1.30–1.31) and was widely stable until 1 year before the index date (OR, 1.29; 95% CI, 1.29–1.30) (Table 4, Fig. 2a).

### Further risk factors and comorbidities

The neuropsychiatric risk factors anxiety, delirium, mental retardation, mood disorder, bipolar disorder, and personality disorder due to brain disease had an increased OR 5 to 10 years before the index date that further increased until 1 year before the index date (Table 4, Fig. 2b). The largest effects were present for delirium and mental retardation at all time points (Table 4, Fig. 2b). The ORs of motor dysfunction and injuries, including abnormalities in gait and mobility, extremity injury, and tremor, was increased from 5 to 10 years before the index date and further increased until 1 year before the index date (Table 4, Supplementary Fig. 2a). Sensory impairments showed increased ORs; ORs decreased between 5 to 10 years and 1 year before the index date for anosmia and visual impairment, while ORs increased between 5 to 10 years and 1 year before the index date for blindness and other sensory disorders (Table 4; Fig. 2b). The OR for sleep disorders was increased at all time points, with an increasing trend from 10 to 1 year before the index date for insomnia and other sleeping disorders and a decreasing trend for restless legs syndrome and sleep apnea (Table 4, Fig. 2b). Patients with migraine had an increased risk of dementia 5 to 10 years before the index date, but with a decreasing trend; thus, 1 year before the index date, patients with migraine no longer had an OR significantly different from 1 (Table 4, Fig. 2c). Conditions in the category of autonomic dysfunction and fatigue were associated with an increased OR for dementia at all time points, with an increasing trend for bladder disorder, constipation, dizziness, and hypotension; a decreasing trend for sexual dysfunction; and a peak 2 to 4 years before the index date for fatigue (Table 4, Supplementary Fig. 2a). Increased ORs were also found for all conditions at all time points in the category of vascular diseases and metabolic disorders, with an increasing trend for atrial fibrillation and cerebrovascular disease and a decreasing trend for carotid stenosis, hypercholesterolemia, and ischemic heart disease until 1 year before the index date (Table 4, Supplementary Fig. 2a). For infections, all categories had increased ORs, with an increasing trend over time for all conditions, including acute upper respiratory infection, antibiotic resistance, cystitis, pneumonia, and sepsis (Table 4, Supplementary Fig. 2a). The OR of gastrointestinal disorders was increased in all periods, with stable effects for Crohn’s disease, duodenal ulcer, reflux, and gastric ulcer; a slightly increasing trend for gastritis; and a slightly decreasing trend for ulcerative colitis (Table 4, Supplementary Fig. 2a).

The general symptoms and conditions included osteoarthritis and other rheumatoid arthritis with an increasing OR toward the index date and unspecific pain with a decreasing OR toward the index date (Table 4, Supplementary Fig. 2a). Cancer patients had a lower risk for dementia starting at year 5 before the index date, with the lowest risk occurring one year before the index date (Table 4, Fig. 2c).

All comorbidities, including Parkinson’s disease, schizophrenia, psychosis, and multiple sclerosis, had increased ORs, with an increasing trend toward 1 year before the index date (Table 4, Supplementary Fig. 2b).

### Prodromal conditions

Cognitive impairment, memory impairment, conduct disorder, and senility were associated with an increased OR for dementia 5 to 10 years before the index date and a further increase in the OR until 1 year before the index date (Table 4, Supplementary Fig. 2b).

## Discussion

Here, we considered 63 risk factors, prodromal features and comorbidities of dementia in a case–control study of more than 1.6 million incident cases with dementia and more than 3.2 million nondemented controls over 10 years preceding the index date in 2022. The data were obtained from claims data diagnoses in primary care and specialized care practices. Almost all studied factors had ORs above 1 for all time points before the index date. The exceptions were unilateral hearing impairment, visual impairment, sexual dysfunction, migraine and other rheumatoid arthritis, with an OR of approximately 1 in year 1 before the index date; Crohn’s disease, with a significantly increased risk of dementia only in year 1 before the index date; and cancer, with a reduced risk of dementia for all time points.

The highest ORs (> 2.0) were associated with (in descending order) delirium, memory impairment, mental retardation, personality and behavioral disorders due to brain disease, damage and dysfunction, sensory disorders, schizophrenia, psychosis, conduct disorder, bipolar disorder, cognitive impairment, alcohol abuse or dependence, Parkinson’s disease, and epilepsy. Memory and cognitive impairment and, to some degree, delirium may be considered prodromal features of dementia [8, 10, 14], accounting for the high risk carried by these factors. The assumption that these factors represent prodromal features is consistent with the steep increase in the OR for these factors between year 10 and year 1 before the index date. We found strong associations with personality and behavioral disorders, conduct disorder, and mental retardation, which are less well established. Conduct disorder (ICD-10 F91 and F92) is typically a diagnosis of childhood and adolescence [38]. Our data showed a low prevalence of 0.31% in controls and 0.75% in cases. The association of a diagnosis of conduct disorder with dementia suggests that it was primarily used to code newly acquired changes in social behavior rather than behavioral disorders that have existed since childhood. This is also illustrated by the large jump in the OR between year 2 and year 1 before the index date. This finding supports the notion that the diagnostic code conduct disorder indicates prodromal features of dementia affecting social behavior. Similarly, personality and behavioral disorders due to brain disease showed a steep increase toward the index date and are likely to represent prodromal features of dementia. Mild to moderate mental retardation is known to increase the risk of dementia not only by lowering the threshold for manifestation of cognitive impairment [39] but also by harboring biological drivers of specific dementia diseases, such as cerebral amyloidosis in Down syndrome, as a risk for presenile-onset AD dementia [40]. The prevalence of mental retardation in the control group was 0.13%, which is lower than the expected 1 to 10% from population-based prospective studies [41]. However, mild mental retardation is typically not diagnosed in clinical care, and the rates of diagnosis of mental retardation decline after early adulthood [41].

Schizophrenia and psychosis may be both risk factors for and comorbidities of dementia. Late-onset cases may even represent prodromal features [42]. This has been described in the context of Lewy body dementia [43], but schizophrenia and behavioral variant frontotemporal dementia have also shown some common pattern of brain atrophy [44]. Neuroleptic medications may mediate some of the risk of schizophrenia and psychosis [45], including dopaminergic dysfunction with typical antipsychotics and increased risk of metabolic syndrome and cardiovascular disease with both typical and atypical antipsychotics. We cannot disentangle these different contributions on the basis of the health claims data, but the jump in the OR between year 2 and year 1 before the index date would be consistent with the assumption of a prodromal feature of dementia for at least some of the diagnoses.

Epilepsy and Parkinson’s disease are both comorbidities and risk factors for dementia, with a greater risk of epileptic seizures in people with neurodegenerative dementia [46] and a greater risk of cognitive decline in people with late-onset epilepsy [47]. The association of Parkinson’s disease with dementia is well established [48]. The link between bipolar disorder and dementia is less well documented; however, several studies have reported an increased risk of dementia in people with bipolar disorder [49, 50]. This effect was even present when considering evidence for a protective effect of lithium for AD dementia [50, 51]. The prevalence of bipolar disorder in the controls was 0.25%. A US survey reported a 12-month prevalence of 0.4% for bipolar I disorder in the 65 + age group [52]. A higher prevalence of bipolar II disorder is expected, but this disorder is less likely to be coded in health claims data. Our prevalence data are in a similar range to those of a previous study [52], suggesting that bipolar disorder may be a risk factor rather than a prodromal feature or comorbidity. However, the linear increase in the prevalence of bipolar disorder toward the index date may be partly due to its being coded as a prodromal feature of dementia. Other sensory disorder had a high OR as well. The OR increased sharply from 2.38 to 3.8 between 2 and 1 year prior to the index date. The category is very broad and unspecific, including visual hallucinations that may be a prodromal feature of dementia, particularly of Lewy body dementia but also unspecific sensory symptoms. Unspecific sensory symptoms have already been described in Parkinson’s disease [53, 54]; our data suggest that they may also be a more general feature of prodromal dementia.

In our large case–control study, we were able to confirm the key risk factors identified by the Lancet Commission and WHO consortia, including alcohol abuse or dependence, hearing impairment, depression, hypertension, obesity, nicotine abuse, traumatic brain injury, and dyslipidemia. Notably, individuals with two-sided hearing impairment and unspecified hearing impairment had a greater odds ratio for dementia than those with unilateral hearing impairment. This finding is consistent with a proposed mechanism of action of hearing impairment through social deprivation and increased demands on cognitive reserve [55]. These factors are less pronounced in unilateral hearing impairment patients. For obesity, we found a decreasing trend in the OR toward the index date, consistent with the notion that midlife rather than late-live obesity is a risk factor for dementia [56–58]. Similarly, for hypertension, the OR decreased closer to the index date, consistent with the notion that obesity and hypertension may confer increased risk several years rather than immediately before the onset of dementia. Hypertension was the most common risk factor, with a prevalence of almost 80% in the controls. This makes hypertension a particularly relevant risk factor at the population level. The effect of hypercholesterolemia was small and decreased closer to the index date. It is still debated whether depression should be considered a risk factor or a prodromal feature of dementia. Data from long-term cohorts suggest that depression increases dementia within five to ten years before the onset of dementia, but not when the distance is more than ten years [9, 59], which would suggest that depression is a prodromal feature of dementia. The steady increase in the odds ratio for depression from −10 to −1 year before the index date (Fig. 2a) would be consistent with a prodromal state, but we cannot prove this based on our time frame.

Sleeping disorders are both a risk factor and a prodromal feature of dementia. Among the sleeping disorders, insomnia had the highest OR, with an increase toward the index date. Cases with sleep apnea had only a slightly increased OR, which was unexpected because of the strong mechanistic data for the effect of sleep apnea on AD biomarkers and brain structure and function [60, 61]. Restless legs syndrome (RLS) also conferred a small risk, with an OR between 1.09 and 1.13. RLS has been discussed to be part of the Parkinson spectrum but also to be a separate entity [62]. Our data showing an increased OR that decreased near the index date suggest that RLS is a risk factor rather than a prodromal feature of dementia.

Gastrointestinal diseases were found to be associated with an increased risk of dementia in a prospective cohort study from the UK Biobank [63]. According to our data, Crohn’s disease, reflux disease, ulcerative colitis, gastritis and duodenal and gastric ulcer were associated with a mildly elevated OR for dementia, with the largest effects for duodenal and gastric ulcer, with an OR of approximately 1.2 across all time points. The UK data and our results contrast with those of a case‒control study in 11,000 dementia patients and 45,000 controls in Korea, which showed no increased risk of dementia in people with a history of peptic ulcer [64]. Different ethnic and cultural backgrounds may influence the effect of gastric diseases on dementia risk. Notably, gastrointestinal diseases have received increasing attention since the recognition of the microbiome as a potential protective or risk factor for brain diseases, including dementia [65]. Obviously, our data cannot reveal any underlying mechanisms; however, the consistent findings across all types of gastrointestinal diseases and the stable effect across the different time points suggest that these diseases may be relevant risk factors for dementia, at least in people of Caucasian ethnicity.

According to our data, cancer was the only disease associated with a significantly lower risk of dementia. An inverse association between cancer and dementia risk has been described in two systematic reviews that suggested a lower incidence of cancer in people with Alzheimer’s disease [66, 67]. Interestingly, a lower risk of dementia in women with breast cancer was detectable independently of a potential effect of chemotherapy or other cancer treatments that may have had a negative impact on cognition [68]. Biological factors have been discussed to explain this inverse association of cancer with dementia. Apoptosis in neurodegenerative dementias may be protective against cancer and, by preceding dementia onset for several years, may lead to a lower incidence of cancer in people who later develop dementia. In addition, cancer and neurodegenerative dementia share some common pathways, such as gene expression regulation [69], that may act in opposite directions. Another factor, however, may be selective mortality and a lower dementia risk in cancer survivors. A prospective evaluation of the Framingham Heart Study captured incident cases of both cancer and dementia over a very long observation period and could control for mortality [70]. Nevertheless, the study [70] could not rule out a survivor effect, i.e. that people who had died of cancer before the age of 65 would have had an increased risk of dementia if they had survived.

According to our data, cancer had no effect on dementia risk between 10 and 6 years before the index date but began to have a lower risk only at 5 years before the index date, which increased as the index date approached (Fig. 2c). Obviously, health claims data cannot serve to confirm or exclude mechanisms of an effect. However, the finding of a protective effect starting only 5 years before dementia onset renders effects related to the biology of cancer less likely. One would expect that biological effects, such as cell proliferation or shared gene expression, would exert their effect on a long-term basis and not explain why the effect becomes most pronounced one year before the diagnosis of dementia. A possible explanation would be a reverse diagnostic effect, in which people with prodromal symptoms of dementia are less likely to recognize and report symptoms of cancer, so that the detection rate for cancer is already lower several years before the onset of dementia. An alternative explanation would be a reverse biological effect, in which the apoptosis of neurodegeneration leads to a reduced manifestation or progression of neoplastic lesions. Neither of these explanations can be confirmed or excluded by our data.

### Strengths and limitations

This is one of the largest case–control studies on dementia risk factors to date and is representative of the general population of Germany in primary and specialized care. It included information on the diagnosis of dementia from general and specialist practices. Our study has several limitations. Health claims data can only be used to generate rather than confirm hypotheses about the mechanisms of effects. Routine data are generated in the context of medical care for medical documentation and cost calculation for health insurance companies without scientific grounds for collection. The advantage of this data is the specific personal reference, high case numbers and the possibility of longitudinal studies. Apart from the ICD classification, there is a lack of other information that would enable external diagnosis validation. In addition, health claims data are subject to a potential diagnostic bias that can theoretically operate in two directions. People with one medical diagnosis may be more likely to receive another diagnosis because they may already be under closer medical scrutiny. At the same time, people with dementia or prodromal features of dementia may be more likely to ignore signs of illness and thus be underdiagnosed. For example, restrospective cohort studies found that breast or colon cancer was diagnosed in more advanced stages in people with dementia compared with non-demented elderly [71, 72].

Our analysis cannot accurately distinguish between risk factors and comorbidity or prodromal feature. Therefore, we used this distinction here for pragmatic structuring of a large number of factors rather than as a clear-cut categorization system. Additionally, risk factors may act differentially on different subtypes of dementia. However, the validity of dementia subtype diagnoses in claims data is limited [73], so we avoided further stratification to prevent additional noise in the data.

Furthermore, we are aware that bivariate associations may overestimate the real associations, as risks from several conditions may accumulate in individual patients, likely resulting in inflated ORs. A possible approach to address this would be to adjust the associations for the other factors. A necessary precondition to modeling adjusted effects in this multivariable framework would be researching previous evidence and incorporating knowledge of an assumed causal structure into the design of the statistical models of all 63 potential risk factors and prodromal features of incident dementia. Failure to do so may result in highly biased estimates, especially when one or more variables are the effect of two or more variables on the casual pathway, which often is referred to as collider bias or collider-conditioning bias [74]. Adjusting total effects, however, ie. “the net of all associations of a variable through all causal pathways to the outcome” [75] lied outside of the scope of our study and appeared even infeasible given the high number of factors.

Our study period from 2012 to 2022 covered the years of the COVID-19 pandemic, starting in 2020. A systematic review showed that utilization of outpatient service declined in 2020 in many countries, including Germany. However, reductions were less pronounced for people with severe illness [76]. Our study group published trends in incidence of newly diagnosed cardiac diseases and hypertension during the time period from 2015 to 2021, also using outpatient claims [77]. We observed no trend changes during the years 2020 and 2021 for newly diagnosed cardiac disease. However, incidence of newly diagnosed hypertension showed a disproportionate decline in 2020 but not in 2021. In conclusion, it’s likely that the detection of prevalent disease, particularly less severe conditions, using outpatient claims data in general was hampered by reduced healthcare attendance during the COVID-19 pandemic, especially in 2020. It is not clear whether people with dementia were disproportionately affected by this phenomenon compared to other population groups.

Despite these limitations, our study represents one of the largest datasets to date investigating risk factors for dementia.

## Conclusions

In a large case–control study of more than 1.6 million cases diagnosed with dementia and more than 3.2 million age- and sex-matched controls, we identified a wide range of risk factors that predicted the onset of dementia by 10 years. Here, we could investigate the temporal trajectories of multiple risk factors preceding dementia. On this basis we could identify different patterns of associations of risk factors with a dementia diagnosis. Some factors showed a large increase in risk one year before the index date compared with the previous dates, suggesting that part of this risk represented the diagnosis of a prodromal feature, such as conduct disorder and personality and behavioral disorders due to brain disease. Confirming the expected difference between the risk of bilateral vs. unilateral hearing impairment supports the notion that the effects were not only due to a diagnostic bias in health claims data but at least partly represented specific effects from specific diagnoses. Notably, 23 of the 63 risk factors had an OR of 1.2 or smaller, carrying only little risk as a single factor. Among them were obesity, ischemic heart disease, migraine, pneumonia, and reflux disease. Thirteen factors had ORs above 2, indicating a high risk. These factors included alcohol abuse or dependence, Parkinson’s disease, and epilepsy. Interestingly, the unspecific diagnosis of other sensory disorders had an OR of 2.9, suggesting that nonspecific symptoms that are not easily classified in clinical practice may be associated with a surprisingly high risk of dementia and should stimulate careful clinical follow-up.

## Supplementary Information

Supplementary Material 1.

Supplementary Material 2.

Supplementary Material 3.

## Data Availability

The datasets analyzed during the current study are not publicly available due to data protection regulations by the German Social Security Code (Sozialgesetzbuch V).

## Abbreviations

AD: Alzheimer's disease
CI: Confidence interval
ICD-10-GM: International Classification of Diseases and Related Health Problems
LC: Lancet Commission
OR: Odds ratio
RLS: Restless legs syndrome
SD: Standard deviation
SHI: Statutory health insurance
STROBE: Strengthening the Reporting of Observational Studies in Epidemiology
WHO: World Health Oganization
