# Supplementary material for: Timing of risk factors, prodromal features, and comorbidities of dementia from a large health claims case–control study
Source: Alzheimers Res Ther. 2025 Jan 16;17:22. doi: 10.1186/s13195-024-01662-x (PMC11736938; doi:10.1186/s13195-024-01662-x)
Supplement: Supplementary file 1 — Supplementary Material 1. [file 13195_2024_1662_MOESM1_ESM.docx]

**Supplementary Table 1: Assignment of quarters to years and intervals**

| **End quarter** | **Start quarter** | **year** | **intervals** |
| --- | --- | --- | --- |
| -1 | -4 | -1 | 1 year |
| -5 | -8 | -2 | 2-4 years |
| -9 | -12 | -3 |  |
| -13 | -16 | -4 |  |
| -17 | -20 | -5 | 5-10 years |
| -21 | -24 | -6 |  |
| -25 | -28 | -7 |  |
| -29 | -32 | -8 |  |
| -33 | -36 | -9 |  |
| -37 | -40 | -10 |  |

**Supplementary Figure 1: Interactive graph of prevalence in controls regressed on odds ratios**

The interactive graph plots the prevalence of each condition in the controls against the odds ratio for dementia risk.

**Supplementary Figure 2. Prevalence of risk factors by year before the index date in cases and controls**

**Figure 2a. Prevalence of comorbidities**

**Figure 2b and c. Prevalence of remaining conditions**

Prevalence of each condition (and 95% confidence intervals) by year before the index date in the cases and controls.
