# Supplementary material for: Timing of risk factors, prodromal features, and comorbidities of dementia from a large health claims case–control study
Source: Alzheimers Res Ther. 2025 Jan 16;17:22. doi: 10.1186/s13195-024-01662-x (PMC11736938; doi:10.1186/s13195-024-01662-x)
Supplement: Supplementary file 3 — Supplementary Material 3. [file 13195_2024_1662_MOESM3_ESM.zip › Supplementary figure 2bR.pdf]

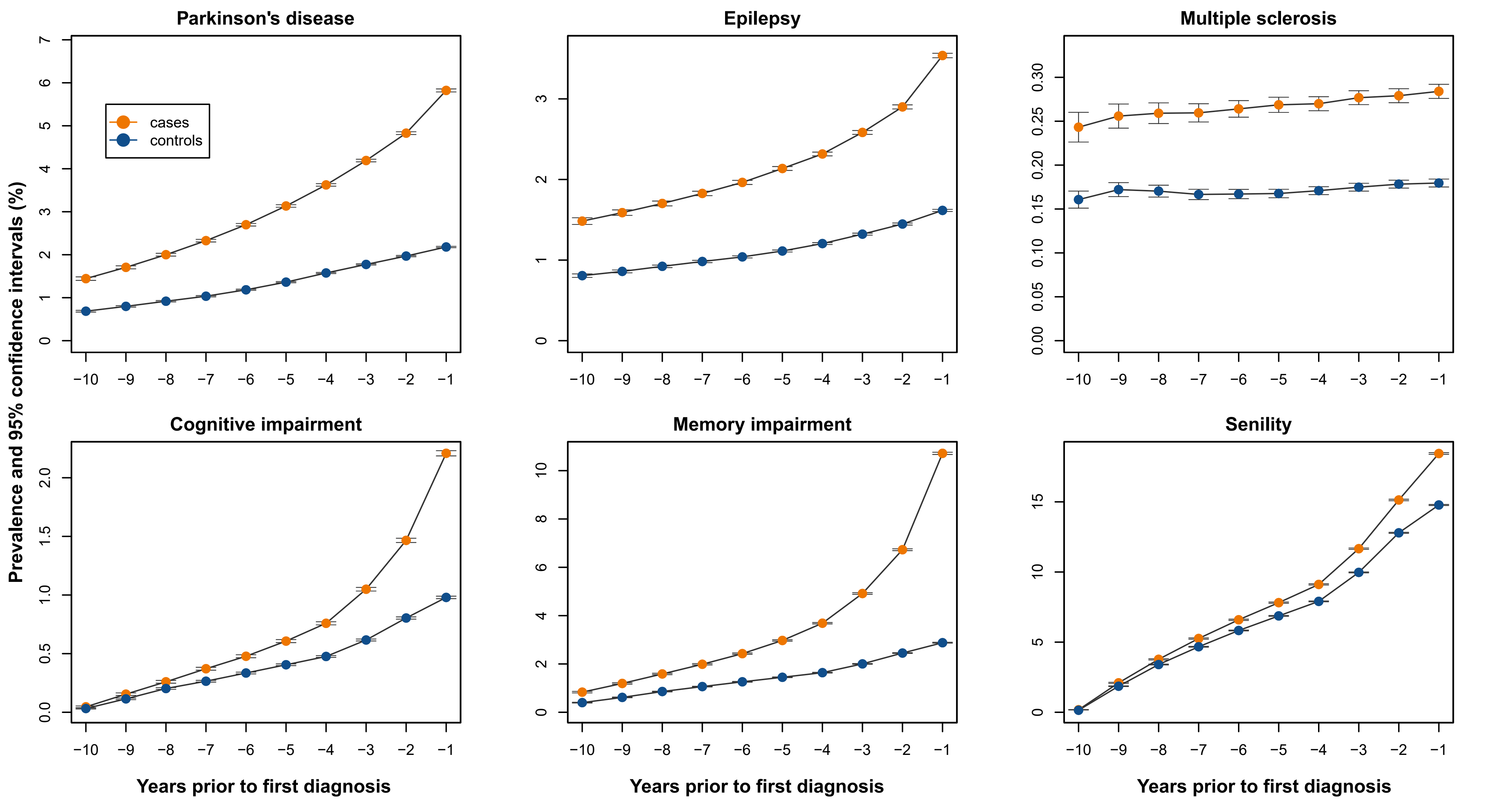

Parkinson's disease
Epilepsy
Multiple sclerosis
Cognitive impairment
Memory impairment
Senility
cases
controls
Prevalence and 95% confidence intervals (%)
Years prior to first diagnosis
−10
−9
−8
−7
−6
−5
−4
−3
−2
−1
