# Supplementary material for: Timing of risk factors, prodromal features, and comorbidities of dementia from a large health claims case–control study
Source: Alzheimers Res Ther. 2025 Jan 16;17:22. doi: 10.1186/s13195-024-01662-x (PMC11736938; doi:10.1186/s13195-024-01662-x)
Supplement: Supplementary file 3 — Supplementary Material 3. [file 13195_2024_1662_MOESM3_ESM.zip › Supplementary figure 2cR.pdf]

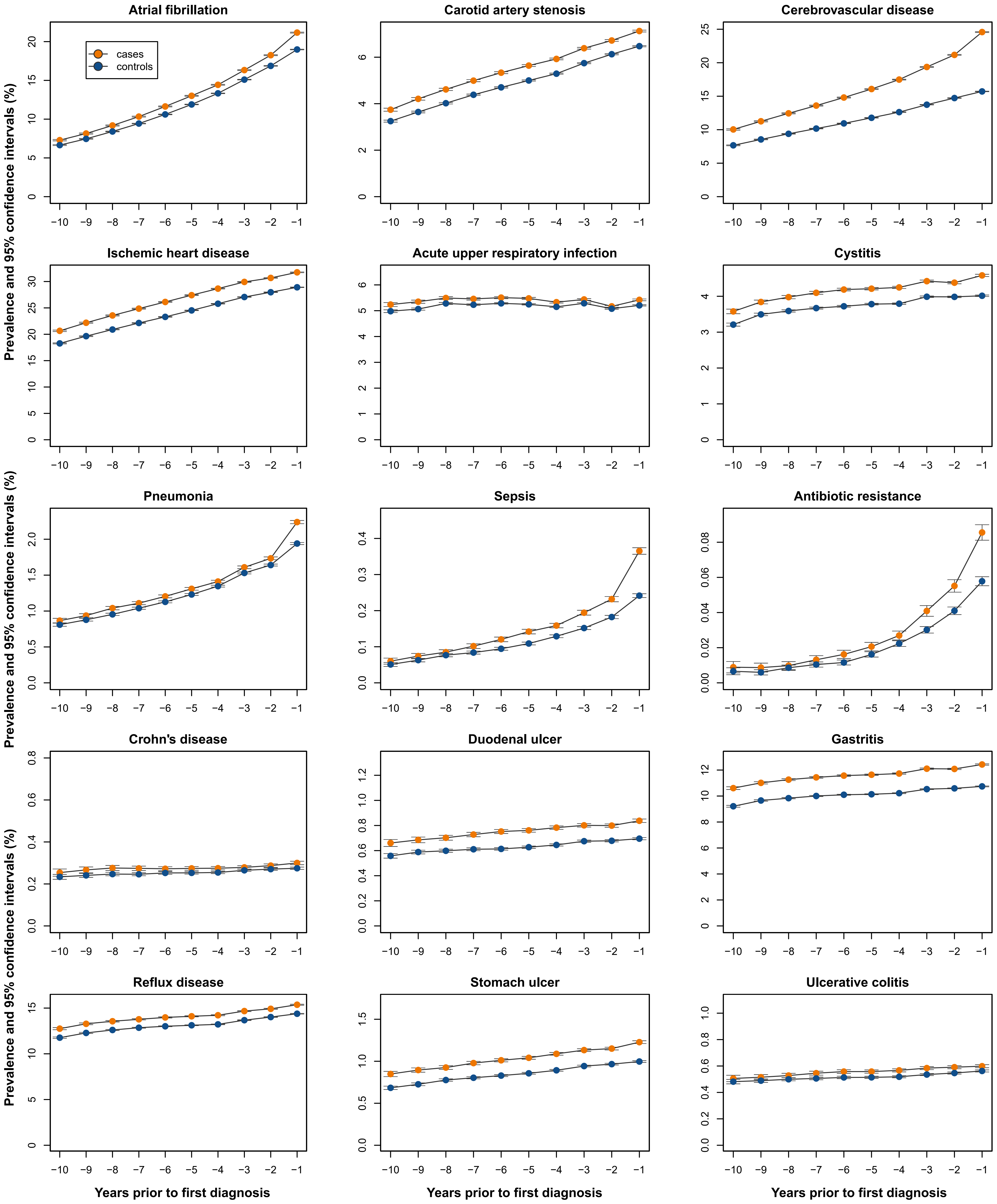
Atrial fibrillation
Carotid artery stenosis
Cerebrovascular disease
Ischemic heart disease
Acute upper respiratory infection
Cystitis
Pneumonia
Sepsis
Antibiotic resistance
Crohn's disease
Duodenal ulcer
Gastritis
Reflux disease
Stomach ulcer
Ulcerative colitis
cases
controls
Prevalence and 95% confidence intervals (%)
Years prior to first diagnosis
